# The effect of an intraoperative, lung-protective ventilation strategy in neurosurgical patients undergoing craniotomy: study protocol for a randomized controlled trial

**DOI:** 10.1186/s13063-018-2447-4

**Published:** 2018-02-02

**Authors:** Liyong Zhang, Wei Xiong, Yuming Peng, Wei Zhang, Ruquan Han

**Affiliations:** Department of Anesthesiology, Beijing Tiantan Hospital, Capital Medical University, No. 6, Tiantan Xili, Dongcheng District, Beijing, 100050 People’s Republic of China

**Keywords:** Randomized controlled trial, Lung protection, Postoperative pulmonary complications, Brain relaxation, Craniotomy

## Abstract

**Background:**

Ventilator-induced lung injury is a major cause of postoperative pulmonary complications (PPCs) in patients undergoing neurosurgery after general anesthesia. However, there is no study on the effect of a lung-protective ventilation strategy in patients undergoing neurosurgery.

**Methods:**

This is a single-center, randomized, parallel-group controlled trial which will be carried out at Beijing Tiantan Hospital, Capital Medical University. Three hundred and thirty-four patients undergoing intracranial tumor surgery will be randomly allocated to the control group and the protective-ventilation strategy group. In the control group, tidal volume (VT) will be set at 10–12 ml/kg of predicted body weight but PEEP and recruitment maneuvers will not be used. In the protective group, VT will be set at 6–8 ml/kg of predicted body weight, PEEP at 6–8 cmH_2_O, and a recruitment maneuver will be used intermittently. The primary outcome is pulmonary complications within 7 days postoperatively. Secondary outcomes include intraoperative brain relaxation, the postoperative complications within 30 days and the cost analysis.

**Discussion:**

This study aims to determine if the protective, pulmonary-ventilation strategy decreases the incidence of PPCs in patients undergoing neurosurgical anesthesia. If our results are positive, the study will indicate whether the protective, pulmonary-ventilation strategy is efficiently and safely used in neurosurgical patients undergoing the craniotomy.

**Trial registration:**

ClinicalTrials.gov, ID: NCT02386683. Registered on 18 October 2014.

**Electronic supplementary material:**

The online version of this article (10.1186/s13063-018-2447-4) contains supplementary material, which is available to authorized users.

## Background

There are at least 234 million patients undergoing major surgeries worldwide each year [1], most of whom need mechanical ventilation when undergoing general anesthesia. Pulmonary complications after mechanical ventilation are the essential reasons for death and disability for patients undergoing general anesthesia [2, 3]. About 25% of patients suffer from a moderate to high risk of postoperative pulmonary complications (PPCs) by undergoing general anesthesia [4, 5]. Several risk factors are related with PPCs including preoperative neurological damage associated with dysphagia, longer duration of surgery, and being placed in the lateral or prone positions. Prevention of PPCs improves the quality of medical care and reduces healthcare costs [5].

General anesthesia and surgical positioning leads to a reduction in functional residual capacity and atelectasis [6, 7]. In the traditional strategy, a tidal volume of 10 to 15 ml/kg is often used to maintain enough gas exchange and intraoperative respiratory dynamics during mechanical ventilation under general anesthesia [8, 9]. However, recent clinical and laboratory researchers have indicated that high-tidal-volume ventilation leads to alveolar over-inflation, partial pulmonary atelectasis, and ventilator-induced lung injury (VILI) [10, 11]. VILI contributes to organ dysfunction mediated by inflammatory factors. Animal experiments have also confirmed that mechanical ventilation with a high tidal volume leads to acute lung injury in healthy lungs [12, 13], stimulation of cytokine production, release of inflammatory substances and inflammatory cell aggregation [14–16].

The protective, lung-ventilation strategy is defined as the combination of low tidal volume (4 to 8 ml/kg per body weight), positive end-expiratory pressure (PEEP, > 12 cmH_2_O, especially > 16 cmH_2_O) and a lung-recruitment method (inspiratory pressure maintained at 30–45 cmH_2_O for 30 to 40 s). Theoretically, low tidal volume prevents alveolar overexpansion [15], and higher PEEP prevents atelectasis [17]. The protective, lung-ventilation strategy has been confirmed to be the optimal mode of ventilation in patients with acute respiratory distress syndrome (ARDS) [18, 19], and reduces morbidity and mortality. Therefore, the protective-ventilation strategy is strongly recommended in acute respiratory distress syndrome (ARDS) guidelines [20]. However, there is little research on the effect of a protective ventilation strategy on high-risk surgical patients under general anesthesia.

Since 2009, some prospective studies have begun to study the effect of lung-protective ventilation in patients without ARDS [21–23]. Several clinical studies have examined the effect of ventilation settings on the inflammatory response, PPCs and postoperative pulmonary function. Despite the variety of surgical procedures, protective ventilation improved the pulmonary inflammatory response and lung function, and reduced the risk of potential oxygenation deficits in healthy patients undergoing general anesthesia. The results suggested that the use of low tidal volumes in non-ARDS patients efficiently and significantly improved the clinical prognosis. Clinical studies in patients undergoing general anesthesia for abdominal surgery [24, 25] as well as meta-analyses and a Cochrane review [26, 27] have shown that PPCs were prevented by a combination of low tidal volume and PEEP. Protective ventilation improved oxygenation and respiratory compliance, and significantly reduced the incidence of VILI.

There are a few studies about the use of the protective, pulmonary-ventilation strategy in neurosurgery. The concern is whether the high-PEEP (>12 cmH_2_O) and lung-recruitment method affect cerebral venous return, decrease brain relaxation and the space for intraoperative operating. However, patients undergoing neurosurgical surgery are often at high risk of PPCs and need particularly close attention. One study showed that high PEEP improved the outcome in a small sample of patients with ARDS after traumatic brain injury, without significantly changing cerebral perfusion and systemic hemodynamic status [28, 29]. When PEEP increased from 5 to 15 cmH_2_O, intracranial pressure (ICP) increased from 15 mmHg to 18 mmHg and cerebral perfusion pressure decreased from 78 mmHg to 72 mmHg, which were all within acceptable limits. However, there is no randomized controlled trial with a large sample to study the effectiveness and safety of the protective pulmonary ventilation strategies, especially in patients with normal lung tissue undergoing neurosurgical surgery for craniotomy.

The objective of this study was to evaluate the influence of lung-protective ventilation (VT 6–8 ml/kg, PEEP at 6–8 cmH_2_O) compared with standard ventilation (VT 10–12 ml/kg, PEEP = 0 cmH_2_O) on the occurrence of PPCs among patients undergoing intracranial surgery.

## Methods

### Study design

**Fig. 1:**
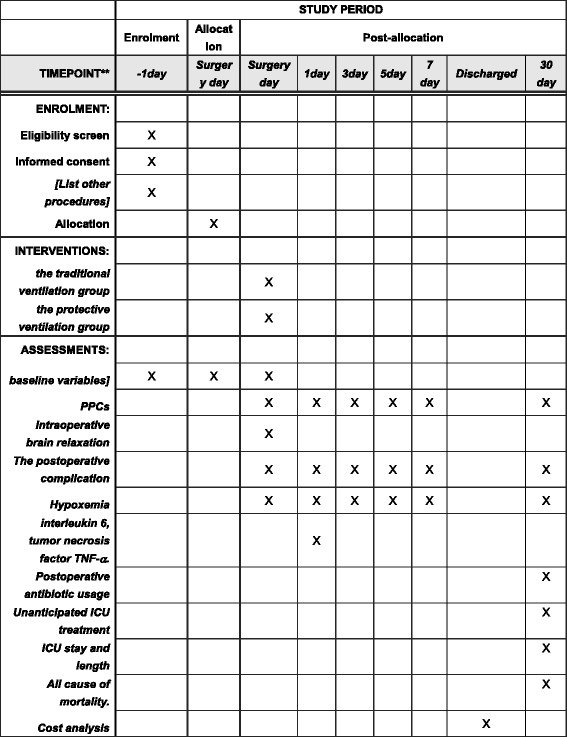
Schedule of enrollment, intervention and assessments

The study is a single-center, randomized, parallel-group controlled trial which is being conducted at Beijing Tiantan Hospital, Capital Medical University. Approximately 3000 patients receive intracranial primary tumor resection at Tiantan Hospital each year, and 200 to 300 patients could meet the inclusion criteria every year. Study recruitment commenced in August 2015. The schedule of enrollment and assessments is as in the Standard Protocol Items: Recommendations for Interventional Trials (SPIRIT) Figure (Fig. 1 and Additional file 1: SPIRIT checklist).

### Randomization and blinding

Randomization will be conducted via a computer-generated randomized controlled table. Patients who meet the criteria will be randomly allocated to the two groups within 24 h before surgery. The allocation ratio is 1:1. Permuted randomization will be used and stratified by age (older or younger than 60 years). The designated staff will perform the allocation sequence which will be involved in patient recruitment. The designated staff assistants will assign participants to interventions. This research staff will implement the allocation sequence through opaque, sealed and stapled envelopes sequentially numbered corresponding to the computer-generated sequence. Corresponding envelopes will not be opened until the enrolled participants complete the trial. The anesthesiologist who is responsible for the anesthesia implementation will not be blinded to the grouping and will not participate in the follow-up visit. However, the neurosurgeon who evaluates brain relaxation will be blinded to the group allocation. The patients and the outcome assessor are all blinded to the grouping.

### Selection and withdrawal of participants

#### Recruitment

Participants will be recruited from the neurosurgical wards and identified by their presence on surgical lists. The investigator informs the participant or the participant’s legal representative of all aspects. The study intervention will be completed immediately after the surgery, but follow-up visits will extend to 1 month after surgery. The medical records will be reviewed following hospital discharge for in-hospital complications and medication usage.

#### Inclusion criteria

1. Patients scheduled to receive elective. intracranial. primary intra-axial tumor resection at Beijing Tiantan Hospital, Capital Medical University who are older than 40 years
2. Glasgow Coma Scale score of more than 8 points
3. Expected operation time more than 4 h
4. PPCs’ risk index grade more than two risk classes (see Table 1) [30]

**Table 1:** Preoperative risk classes of postoperative pulmonary complications (PPCs)

Preoperative risk factor	Point value
Neurosurgery	8
Age	
≥ 80 years	17
70–79 years	13
60–69 years	9
50–59 years	4
Functional status	
Totally dependent	10
Partially dependent	6
Weight loss > 10% in past 6 months	7
History of chronic obstructive pulmonary disease	5
History of cerebrovascular accident	4
General anesthesia	4
Impaired sensorium	4
Blood urea nitrogen level	
< 2.86 mmol/L (< 8 mg/dl)	4
7.85–10.7 mmol/L (22–308 mg/dl)	2
≥ 10.7 mmol/L (≥ 30 mg/dl)	3
Transfusion > 4 units	3
Emergency surgery	3
Steroid use for chronic condition	3
Current smoker within 1 year	3
Alcohol intake > 2 drinks/day in the past 2 weeks	2

Grade 1: 0–15 points; Grade 2: 16–25 points; Grade 3: 26–40 points; Grade 4: 41–55 points; Grade 5: > 55 points
5. Having signed the informed consent form

#### Exclusion criteria

1. Patients with chronic lung disease or pulmonary infection 1 month before the surgery
2. Patients with a history of pulmonary surgery
3. Patients with dysphagia resulting from preoperative cranial nerve damage
4. Patients with large tumors of the skull base who are expected to require a tracheal tube after surgery
5. Body Mass Index more than 35 kg/m^2^
6. Acute respiratory failure (pneumonia, acute lung injury, ARDS)
7. Emergency surgery
8. Sepsis or septic shock
9. Progressive neuromuscular disease
10. Pregnant women
11. Heart failure or with severe heart disease

### Study intervention

#### Ventilation parameter setting during operation

All patients will be randomly allocated to the control group (traditional group) and the protective-ventilation strategy group (protective group) according to the computer-generated random number table. In the control group, VT will be set at 10–12 ml/kg of predicted body weight, with PEEP = 0. The recruitment maneuver will not be used. In the protective group, VT will be set at 6–8 ml/kg of predicted body weight with PEEP at 6–8 cmH_2_O, and the recruitment maneuver will be used intermittently. The predicted body weight of male patients is calculated as 50 + 0.91 × (centimeters of height – 152.4) and of female patients is calculated as 45.5 + 0.91 × (centimeters of height – 152.4) [24]. The lung-recruitment maneuver is maintained as an inspiratory pressure of 30 cmH_2_O for 30 s, after tracheal intubation, at the end of surgery and before extubation, respectively. Using lung-recruitment maneuvers in neurosurgery may lead to brain swelling during the operation and increase the risk of cough. When the dura mater is opened, we will do not use the recruitment method. Before using the recruitment method, we need to ensure adequate anesthesia depth or enough muscle relaxation to avoid cough. All patients will receive volume-controlled mechanical ventilation, fraction of inspiration O_2_ (FiO_2_) < 0.5, I:E = 1:2, the respiratory rate will be adjusted according to blood gas analysis results. PaCO_2_ is maintaining at 30–35 mmHg.

#### Concomitant treatments

Peripheral venous access will be established before induction. Routine monitoring and data collection include non-invasive blood pressure, electrocardiograph, pulse oxygen saturation, end-tidal carbon dioxide pressure, exhaled anesthetic concentration, and Bispectral Index. All patients will be induced with sufentanil (0.2 to 0.3 μg/kg), propofol 2–2.5 μg/ml and cisatracurium (1.5 mg/kg). After tracheal intubation, mechanical ventilation will be performed according to the grouping. Inhalation oxygen concentration is 50%. Anesthesia will be maintained with propofol and sevoflurane and remifentanil (0.1 to 0.2 μg/kg/min). The target MAC of inhaled anesthesia will be controlled below 0.5 MAC. The Bispectral Index will be maintained between 40 and 60. Sufentanil will be administered at a specific time point, such as the moment of scalp incision, before the end of the operation. The mean arterial pressure will be maintained between below 20% and above 10% of the baseline value. Fluid input and urine output will be monitored closely. Peripheral arterial blood will be sampled at the beginning and end of surgery, 1 day after surgery to measure the routine blood count and blood gas analysis.

### Study objective

#### Primary outcome

The primary outcome of the trial is to investigate whether the protective ventilation reduces the incidence of the PPCs within 7 days. The definitions of PPCs will be according to the modified Clinical Pulmonary Infection Score (mCPIS) (see Table 2) [31]. We define no pulmonary infection when the grade is zero. We define this as a pulmonary complication if the grade is greater than zero (1 to 4, indicate to severe). The mCPIS includes symptoms and signs (such as a dry cough and sputum), chest x-ray, blood and sputum laboratory tests, blood gas analysis and body temperature after surgery, etc.

**Table 2:** Modified Clinical Pulmonary Infection Score (mCPIS)

Classification	Grading basis
Grade 1	Dry cough
	Atelectasis: extrapulmonary exclude other causes of body temperature> 37.5°C, or abnormal pulmonary symptoms or signs; radiological examination was normal
	Difficulty in breathing (other causes excluded from the lung)
Grade 2	Cough and sputum don’ts due to other causes (heart failure, etc.)
	Bronchospasm: wheeze, or the original wheeze need treatment
	Hypoxemia
	atelectasis: radiological evidence; body temperature> 37.5°Cor abnormal lung symptoms or signs
	Transient hypercapnia requiring treatment such as naloxone; assisted or mechanical ventilation
Grade 3	Pleural effusion, pleurisy
	Pneumonia, suspected: radiological evidence; no positive bacterial culture results
	Pneumonia, diagnosis: radiological evidence; bacterial culture evidence
	Pneumothorax
	Postoperative reintubation or retention of intubation respiratory support (including noninvasive and invasive) ≤48 hrs.
Grade 4	Respiratory failure: postoperative non-invasive respiratory support ≥ 48 hours; or re-endotracheal intubation ventilator support ≥ 48hrs

* Postoperative hypoxemia diagnostic criteria: suction air PaO_2_ < 60 mmHg, or SpO_2_ < 90%; or PaO_2_/FiO_2_ ≤ 300

* Pneumonia diagnostic criteria: new chest radiograph or progression of infiltrative lung lesions, combined with the following two or more can be diagnosed: ① body temperature ≥ 38.5 °C or < 36 °C; ② WBC > 12 × 109 or < 4 × 109; ③ purulent sputum and/or new or aggravated cough and expectoration

* Atelectasis diagnostic criteria: ① atelectasis by x-ray signs: atelectasis of the lung tissue through decrease in brightness; increased homogeneity of a radiological density; bronchiectasis can be associated with non-homogeneous density (cystic translucent area) in convalescence. Different degrees of volume reduction, subsegmental and distal to the pulmonary atelectasis may have other collateral ventilation routes and volume reduction is not obvious. Leaf segmental atelectasis is generally blunt, triangular, wide and face towards the diaphragmatic pleural surface, the tip pointing to the hilum, in a fan, triangle, band, circle, etc. ② Computed tomography (CT) imaging above costophrenic angle 1 cm

* Systemic inflammatory response syndrome (SIRS) diagnostic criteria: two or more of the following clinical manifestations: ① body temperature > 38 °C or < 36 °C; ② heart rate > 90 beats/min; WBC > 12 × 109 or < 4 × 109 or myeloblast count > 10%

Diagnosis of sepsis: ① systemic infection: positive microbial blood culture, or tissue infection or evidence of abscess formation (such as: pneumonia, peritonitis, urinary tract infection, central venous catheter infection, soft tissue infections); ② at least two SIRS criteria

* Criteria of diagnosis of severe sepsis: sepsis, combined with at least one organ failure, hypotension or hypoperfusion

* Diagnostic criteria for Chinese toxic shock syndrome: infection-induced hypotension, although the volume of treatment but there are still important organs and tissue hypoperfusion

#### Secondary outcome

The secondary outcomes are as follows:

1. Intraoperative brain relaxation. Brain relaxation will be scored by the neurosurgeon after opening the cranium and before opening the dura. They will use a 4-point scale: 1, completely relaxed; 2, satisfactorily relaxed; 3, firm brain; 4, bulging brain
2. The airway peak pressure at different time points during operation, such as skin incision, drilling, opening the dura, tumor resection, hemostasis, suturing the dura, the end of surgery
3. The postoperative complications within 30 days including:
  - Surgical complications, including intracranial infection, incision infection, cerebral edema or bleeding requiring re-operative surgery
  - PPCs
  - Systematic complications (systemic inflammatory response syndrome; septic shock, etc.)
  - Death
4. Postoperative gas exchange disorder (hypoxemia) within 30 days: when the patient breathes air, partial pressure of oxygen (PaO_2_) less than 60 mmHg, or Pulse Oxygen Saturation (SpO_2_) less than 90%; or PaO_2_/FiO_2_ less than 300.
5. Peripheral blood inflammatory response indicators on the first postoperative day: interleukin-6, tumor necrosis factor TNF-α
6. Postoperative antibiotic usage within 30 days
7. Unanticipated intensive care unit (ICU) treatment within 30 days
8. ICU stay and length of hospital stay within 30 days
9. All-cause of mortality at 30 days
10. Cost analysis: data on the cost of treatment will include standardized costs for physiotherapy, neurosurgery, anesthesia and postoperative care. Data will be presented regarding total non-operative costs, costs per day.

#### Reporting of adverse events

All adverse events will be recorded and closely monitored until resolution or stabilization or until it has been shown that potential conflicts of interest regarding the study treatment are not the cause of the event. In the event of any serious adverse event, it will be immediately reported to the Endpoint Adjudication Committee, which will determine the severity and causality of the adverse events. The chief investigator will be responsible for all adverse event reporting.

### Withdrawal from the trial

We will consider patient withdrawal from the trial if the following conditions occur: (1) severe brain swelling during the operation; (2) the patient has a cough during surgery; (3) the patient has persistent hypotension and circulatory instability.

### Data collect and management

We can obtain all the patient information through the electronic medical record system. We also obtained the consent of the neurosurgeon and the radiologist who will help us make the neurological diagnosis. All personal information will be collected through the hospitalized medical records by a member of the research team and be kept strictly confidential for research purposes only. The research team members will be responsible for maintaining personal data. Only the primary investigator and the designated researcher can obtain interim results and final test data.

### Data Monitoring Committee (DMC)

The project will be monitored by a Data Monitoring Committee (DMC) composed of specialists in anesthesiology, ethics, statistics and methodology. The DMC will audit through regular interviews or telephone calls.

### Sample size and justification

We calculated the sample size through the website http://www.sample-size.net/sample-size-proportions/.

The incidence of pulmonary complications was 36% after about 2 h of abdominal surgery within 7 days [24], in which PPCs were assessed through the mCPIS. When the duration of intracranial surgery was more than 300 min, the probability of PPCs was 28.4% [32], in which PPCs were defined as pneumonia, tracheobronchitis, atelectasis and bronchoconstriction. However, if the PPCs were defined by using the mCPIS scale from grades 1 to 4, the incidence of PPCs would significantly increase to more than 28.4%. In our study, the duration of surgery is expected to be more than 5 h and the PPCs are evaluated by the mCPIS, So, we estimate that the incidence of PPCs would be greater than 36%, and define 40% as the incidence of the PPCs.

Protective lung ventilation leads to the incidence of pulmonary complications decreasing from 36% to 17.5% in patients undergoing abdominal surgery [24], which was consistent with the Cochrane review [27]. Therefore, we also define the effect size of the protective lung ventilation as 18.5%.

So, we set P0 = 40%, P1 = 40% -18.5% = 21.5%, *α* = 0.05 and *β* = 0.1. The allocation ratio is 1:1. The sample size is 278. Taking account the dropout rate of 20%, the total sample size is 334 cases [33, 34].

### Statistics

The SPSS 19.0 software package for Windows (SPSS, Inc., Chicago, IL, USA) will be used for all statistical analyses. The primary outcome will be expressed as the number of patients (percentage) or median (interquartile range (IQR)), and analyzed by using the chi-square (χ^2^) and Fisher’s exact tests. The difference in severity of pulmonary complications between the two groups will be made based on the patient grading score of the two groups. We will use the Mann-Whitney *U* test for analysis. Brain relaxation will use the Mann-Whitney *U* test for analysis. The airway peak pressure, cost analysis, ICU stay and length of hospital stay, postoperative antibiotic usage, and peripheral blood inflammatory response indicators will use a one-way analysis of variance. The incidence of postoperative complications within 30 days, hypoxemia, unanticipated ICU treatment and all-cause mortality at 30 days will be expressed as the number of patients (percentage) or median (interquartile range (IQR)), and analyzed by using the chi-square (χ^2^) or Fisher’s exact tests.

When the follow-up visits of 150 participants are completed (estimated to occur after 18 months of recruitment), the interim analysis will be conducted to evaluate the efficacy of the primary outcome. The *p* value for the analysis will be set at *p* < 0.001 using the alpha-sparing technique (O’Brien-Fleming) for benefit or harm.

## Discussion

This study is a randomized controlled trial on the effect of protective lung ventilation on PPCs in patients undergoing neurosurgery. There has been little attention paid so far to patients undergoing craniotomy. However, the incidence of neurological PPCs is relatively high. Qaseem et al. [35] reported that the risk of PPCs increased when surgical duration is more than 4 h. The incidence of PPCs was 28.4% (20.2–37.9%) in patients with neurosurgery lasting for longer than 300 min [32].

PEEP is proposed to reduce the incidence of postoperative respiratory complications. PEEP may prevent atelectasis, and reduce the risk of VILI. Higher PEEP use in neurosurgery is relatively limited because higher PEEP may lead to increase airway pressure, decreased cerebral venous return and intraoperative operating space. Recent research has provided compelling evidence that lung-protective mechanical ventilation using lower tidal volumes, moderate PEEP (6–8 cmH_2_O) and lung-recruitment maneuvers were associated with improved functional or physiological and clinical postoperative outcome in patients undergoing abdominal surgery [36]. We also observed peak airway pressure less than 20 cmH_2_O in the pilot study and adequate operating space was not affected. Therefore, we will use moderate PEEP (6–8 cmH_2_O) to avoid the effects of the higher PEEP on intracranial pressure (ICP) in this study.

Whether PEEP can be safely used in craniotomy is a critical issue. So, the brain relaxation evaluations will be performed before dural incision. If intracranial pressure increases sufficient to affect the operation by using PEEP, we will abandon the case and change the parameter of ventilation. This case will be reported to the primary investigator.

The study is a prospective, randomized controlled, double-blind trial. This study aims to determine whether the protective pulmonary ventilation can be efficiently and safely used in neurosurgical patients undergoing the craniotomy. If we can prove that protective lung ventilation can reduce the incidence of PPCs in patients undergoing craniotomy, it will improve the prognosis of neurosurgical patients and decrease medical costs.

### Trial status

The study was also registered on the registry website http://clinicaltrails.gov/ with the registration number NCT02386683 on 18 October 2014. The study began on 1 October 2015, and the planned completion date will be March 2018. Trial status was currently recruiting.

## Additional file

Additional file 1: SPIRIT Checklist. (DOCX 41 kb)

## Abbreviations

PPCs: Postoperative pulmonary complications
VT: Tidal volume
ICP: Intracranial pressure
VILI: Ventilator-induced lung injury
PEEP: Positive end-expiratory pressure
ARDS: Acute respiratory distress syndrome
PaO_2_: Partial pressure of oxygen
SpO_2_: Pulse oxygen saturation
FiO_2_: Fraction of inspiration O_2_
